# Multidisciplinary inpatient rehabilitation for older adults with COVID-19: a systematic review and meta-analysis of clinical and process outcomes

**DOI:** 10.1186/s12877-023-04098-4

**Published:** 2023-06-27

**Authors:** Aoife McCarthy, Rose Galvin, Frances Dockery, Kara McLoughlin, Margaret O’Connor, Gillian Corey, Aoife Whiston, Leonora Carey, Fiona Steed, Audrey Tierney, Katie Robinson

**Affiliations:** 1 School of Allied Health, Faculty of Education and Health Sciences, Post Graduate Member HRI, University of Limerick, Limerick, Ireland; 2 School of Allied Health, Faculty of Education and Health Sciences, Ageing Research Centre, Health Research Institute, University of Limerick, Limerick, Ireland; 3 Department of Geriatric and Stroke Medicine, and Integrated Care Team for Older People North Dublin, Beaumont Hospital, Dublin, Ireland; 4 Department of Occupational Therapy, Beaumont Hospital, Dublin, Ireland; 5 Department of Ageing and Therapeutics, University Hospital Limerick, Limerick, Ireland; 6 School of Medicine, Faculty of Education and Health Sciences, University of Limerick, Limerick, Ireland; 7 Post Doctoral Researcher, Ageing Research Centre, University of Limerick, Limerick, Ireland; 8 UL Hospitals Group, University Hospital Limerick, Limerick, Ireland

**Keywords:** Systematic review, Older adults, Rehabilitation, Outcomes, COVID-19

## Abstract

**Background:**

Older adults are at increased risk for disease severity and poorer prognosis following COVID-19 infection. The aim of this systematic review and meta-analysis is to explore the impact of multidisciplinary rehabilitation in the acute or post-acute hospital setting for older adults with COVID-19.

**Methods:**

The Cochrane library, EMBASE, Cinahl and Medline (via EBSCO), PubMed, and Web of Science were systematically searched in June 2022 and a repeat search was completed in March 2023. Screening, data extraction and quality appraisal were conducted independently by two reviewers. Studies reporting outcomes for older adults following multidisciplinary rehabilitation (provided by two or more Health and Social Care Professionals) were included. Both observational and experimental study designs were included. The primary outcome was functional ability. Secondary outcomes included discharge disposition, acute hospital and rehabilitation unit length of stay, mortality, primary and secondary healthcare utilisation, and long-term effects of COVID-19.

**Results:**

Twelve studies met the inclusion criteria, comprising a total of 570 older adults. Where reported, older adults stayed in the acute hospital for a mean of 18 days (95%CI, 13.35- 23.13 days) and in rehabilitation units for 19 days (95%CI, 15.88–21.79 days). There was a significant improvement in functional ability among older adults with COVID-19 who received multidisciplinary rehabilitation (REM, SMD = 1.46, 95% CI 0.94 to 1.98). The proportion of older adults who were discharged directly home following rehabilitation ranged from 62 to 97%. Two studies reported a 2% inpatient mortality rate of older persons during rehabilitative care. No study followed up patients after the point of discharge and no study reported on long term effects of COVID-19.

**Conclusions:**

Multidisciplinary rehabilitation may result in improved functional outcomes on discharge from rehabilitation units/centres for older adults with COVID-19. Findings also highlight the need for further research into the long-term effect of rehabilitation for older adults following COVID-19. Future research should comprehensively describe multidisciplinary rehabilitation in terms of disciplines involved and the intervention provided.

**Supplementary Information:**

The online version contains supplementary material available at 10.1186/s12877-023-04098-4.

## Background

In March 2020, a global pandemic was declared with the emergence of COVID-19, an infectious disease, viral by aetiology and caused by the SARS-CoV-2 virus [1]. As of the 17^th^ of March 2023, the World Health Organisation (WHO) reported 760,360,956 confirmed cases and 6,873,477 deaths globally [2]. Common symptoms of COVID-19 include fever, dry cough, and fatigue; less commonly people experience headache, dizziness, abdominal pain, nausea, and vomiting [3].

Older age and male gender place people at higher risk for disease severity [3–6] and a poorer prognosis [4, 7]. Those with other underlying health conditions namely cancer, obesity, chronic kidney disease, chronic lung disease, cystic fibrosis, dementia, diabetes, people with disabilities, heart conditions, HIV infection, and those who are immunocompromised are also at greater risk of severe illness [8–10]. Given that over 50% of those aged over 65 have two or more chronic health conditions [11], it would suggest that older persons are at significant risk for COVID-19 disease severity.

Between 13.9 and 43% of patients infected with COVID-19 develop long term symptoms, with fatigue and memory difficulties or brain fog amongst the most common [12, 13]. Additionally, the quality of life (QOL) of those post COVID-19 is significantly impacted regardless of the time since discharge or recovery and older age and co-morbidities are among the most frequently reported factors associated with low levels of QOL post COVID-19 [14]. Worse mobility and functional outcomes have also been identified in older adults admitted to hospital due to COVID-19 [15] and in older adults with mild to moderate COVID-19 who did not require hospitalisation [16].

In the early months of the pandemic there was a dearth of literature describing the rehabilitation needs of people recovering from COVID-19 and the efficacy of interventions [17]. Since this time, the body of evidence has grown significantly to include longitudinal studies exploring clinical progression, symptoms, and rehabilitation recommendations [18–20].

The WHO’s living guideline on the clinical management of COVID-19 recommends screening for rehabilitation needs throughout the recovery process [21] and both the WHO guidelines and the National Institute for Health and Care Excellence (NICE) guidance document for the management of Long Covid [22] recommend multidisciplinary input given the virus’ impact on several body structures and functions, and its long-term sequelae. These guidelines reflect other COVID-19 rehabilitation guidelines developed for clinicians of specific disciplines [23–27] and the European Geriatric Medicine Society (EuGMS) guidance [28]. The use of comprehensive geriatric assessment, long term follow-up and ongoing monitoring of patients following discharge from rehabilitation for COVID-19 is also advised by EuGMS, with suggested time points of 6 weeks and 6 months [28].

Studies have evaluated multidisciplinary team (MDT) rehabilitation for various groups including adults with severe-to-critical illness in intensive care units [29] and those adults post intensive care [30]. Older adults face increased risks for COVID-19 severity and poorer prognosis. While the literature supports multidisciplinary rehabilitation for adults hospitalised with COVID-19, little is known yet about how MDT rehabilitation in this group impacts outcomes. To date, there are no randomised controlled trials or analytical cohort studies published exploring the effect of MDT rehabilitation on older adult outcomes following hospitalisation for COVID-19. However, several observational studies have described the rehabilitation outcomes of older adults with COVID-19 following MDT intervention. There is a need to profile the clinical characteristics, functional and process outcomes of older adults who have undergone MDT rehabilitation in the acute or post-acute inpatient hospital setting to inform the development and response of services in the future and to guide the development of trial studies. This systematic review aims to explore and synthesise the totality of evidence regarding the outcomes of older adults with COVID-19 who have undergone MDT intervention in the acute or post-acute inpatient setting. The author hypothesises that older adults with COVID-19 will have improved function following completion of MDT rehabilitation.

## Methods

### Study design

The conduct and reporting of this systematic review of observational studies is in accordance with the Meta-analysis Of Observational Studies in Epidemiology (MOOSE) guidelines [31], see Additional file 1. The protocol for this systematic review has been registered on the PROSPERO register (PROSPERO ID = CRD42022341365).

### Search strategy

The searches were conducted on the 1^st^ of June 2022 of the following databases: Cochrane library, EMBASE, Cinahl and Medline (via EBSCO), PubMed, and Web of Science by the first author. Reference lists of eligible studies were also checked. Literature was limited to publications from March 2020 to the date of search completion and limited to English language full text. The search was completed by AMC, Master of research candidate at the University of Limerick. A repeat search was conducted on the 17^th^ of March 2023 to identify additional papers published between the initial and repeat search date.

The following MeSH terms and associated keywords covering three concepts were used;

- COVID-19
- Multidisciplinary rehabilitation
- Hospital setting

Appropriate synonyms were compiled to identify all appropriate studies. See Additional file 2 for search terms and synonyms.

### Eligibility criteria

Studies meeting the following criteria were included:

- Population: Older adults (with mean or median age of 65 or greater) with a diagnosis of COVID-19.
- Study design: Prospective and retrospective descriptive cohort studies, comparison groups of experimental studies including randomized controlled trials, quasi randomized studies or controlled before after studies, case series (with more than 1 participant), and the ‘cases’ in case control studies.
- Intervention: multidisciplinary (MDT) rehabilitation provided by two or more Health and Social Care Practitioners (HSCP) including but not limited to the following disciplines in the inpatient setting: Occupational Therapy, Physiotherapy, Speech and Language Therapy, Human Nutrition and Dietetics, Psychology and/or Medical Social Work.

### Outcomes

The primary outcome for the study was any validated measure of functional ability that reflect activity limitations and participation restrictions in keeping with the International Classification of Functioning e.g., Barthel Index, or Functional Independence Measure.

Secondary outcomes included:

- Discharge disposition e.g., discharge directly home, long term care, transitional care, and/or to the care of a family member
- Hospital length of stay (LOS)
- Mortality
- Primary/Community and secondary healthcare utilisation (unplanned ED return, unscheduled hospital admission)
- Long term effects of COVID-19 i.e. signs and symptoms reported during the post COVID-19 phase for example fatigue, headache, attention disorder, hair loss and dyspnoea [32].

Studies were excluded if they met any of the following criteria:

- Population: Persons with COVID-19 with mean or median age of < 65 years.
- Study design: The control arm of experimental or analytical observational studies where MDT intervention has not been implemented, and cross-sectional studies.
- Intervention: Studies reporting outcomes following uni-disciplinary interventions, pulmonary rehabilitation only or papers describing medical interventions only. Studies reporting outcomes following rehabilitation only in the Intensive Care Unit were also excluded.

### Data extraction

Studies obtained through the search strategy were reviewed and duplicates removed in Endnote. Remaining studies were then exported to Rayyan for initial screening by the first author (AMC). Rayyan is a web-based platform that facilitates the methodical and efficient screening of search results by title and abstract. It allows researchers to allocate labels to explain reasons for exclusion facilitating transparency in the systematic review process [33]. One third of included articles were independently reviewed by another author (RG). Following the initial screening, full text articles were obtained and screened for eligibility by two members of the research team (AMC & RG). Disagreement was resolved through review by a third review team member (KR). Where information relating to inclusion and exclusion criteria was ambiguous or not reported in an article, the authors were contacted by email to screen for eligibility.

Data were extracted from included studies by one reviewer (AMC) using a custom template. The following data were extracted: Author, year of publication, country, methodology/ study design, population (including patient demographics and baseline characteristics where applicable), interventions received, and outcomes measured. A quality check of 20% of the data extraction was completed by a second independent reviewer (RG).

### Quality assessment

The methodological quality of included studies was assessed independently and in duplicate by two reviewers (AMC, RG). The CASP critical appraisal tool for cohort studies [34] and the JBI critical appraisal tool for case series [35] were applied as appropriate. Disagreements regarding bias were resolved by a third reviewer (KR). GRADE analysis was applied to the primary outcome of functional ability to evaluate the quality of evidence [36].

### Statistical analysis

Statistical analysis was performed using Review Manager Software (version 5.4) for meta-analysis. For the primary outcome of functional status, the mean and standard deviation values for the MDT group were extracted at baseline and post MDT rehabilitation. In instances where the mean and standard deviation (SD) were not available, the median was used as a proxy for the mean and a multiple of 0.75 times the interquartile range (IRQ) or 0.25 the difference in the range [37]. In studies that assessed the same construct but used a different validated outcome measure to report the construct, the exposure (MDT rehabilitation) effect was determined using the standardised mean difference (SMD). In studies that measured the same outcome using the same scales, the mean difference (MD) was used. The standard error (SE) was calculated using the SD divided by the square root of the number of values in the data set (n)**.** For all outcomes, the denominator in each group was considered as the number of participants allocated to that group at baseline.

We assessed clinical variation across the studies by exploring the characteristics of participants, the content and duration of the MDT intervention, outcome measures administered and timing of outcome assessments. Statistical heterogeneity was examined by visual inspection of the forest plots and using the Chi^2^ statistic and the I^2^ test. As strict thresholds for interpreting I^2^ are not recommended, we interpreted the I^2^ statistic using the approximate guide by Deeks and colleagues [38]. Furthermore, to explore potential explanations of heterogeneity, moderator analysis was conducted where sufficient data was available. For example, random effects meta-regression was conducted when ≥ 10 studies reported a continuous moderator variable—age, gender, length of stay, and number of health and social care professional disciplines. In instances where there was considerable variation in the results or where there was not enough data available to conduct a meta-analysis, we opted for a narrative summary of the outcomes of interest.

## Results

### Flow of studies in the review

**Fig. 1:**
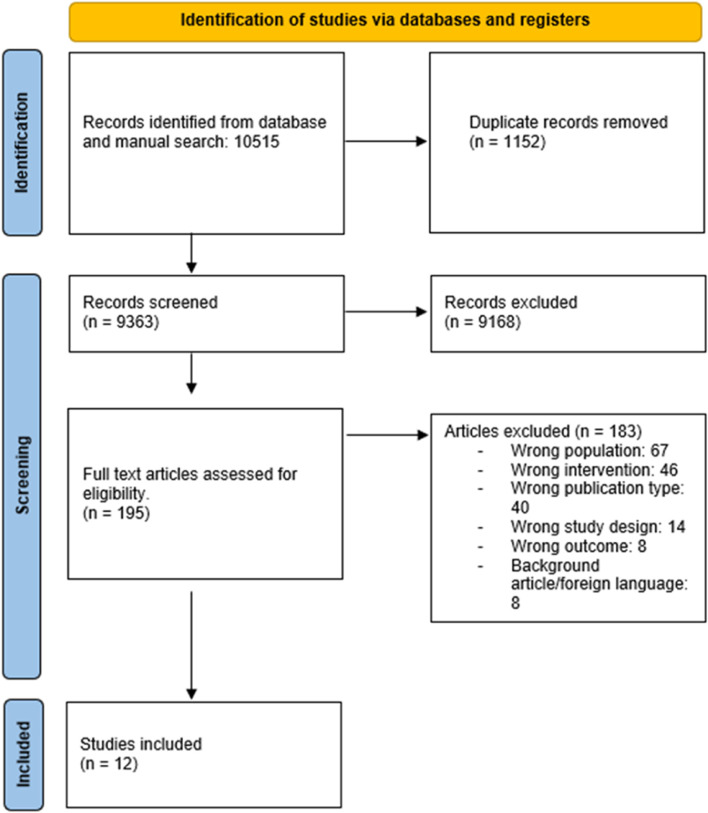
PRISMA (Preferred Reporting Items for Systematic Reviews and Meta-Analyses) flow diagram of included studies

Figure 1 displays the flow of studies in the review. A total of 10,515 studies were identified across the database searches, 9168 were excluded on the basis of title/abstract screening and 195 full text articles were reviewed. Ultimately 12 articles were deemed eligible for inclusion.

### Study and patient characteristics

Twelve studies met the criteria for this systematic review. Four studies were conducted in the United States [39–42], two in Italy [43, 44], two in Switzerland [45, 46], and one study in Canada [47], Romania [48], Taiwan [49] and France [50]. Six studies in the review were published in 2021 [39, 40, 43, 44, 47, 50]. Four were published in 2022 [41, 45, 48, 49]. Two papers were published in 2023 [42, 46]. The total number of participants from included studies was 570. Nine out of 12 studies reported the age of the cohort as a mean (65 to 85.33 years) [39–42, 44–46, 48, 49]. The remaining three studies reported a median age of 65 to 75 [43, 47, 50]. Older adults required ICU admission in seven out of 12 studies ranging from 23 to 100% of their total cohort [41, 43, 44, 46, 47, 49, 50].

### Rehabilitation programme

Each paper described MDT rehabilitation which included 2 or more HSCP disciplines including Physiotherapy (PT), Occupational Therapy (OT), Speech and Language Therapy (SLT), Psychology, Social Work, Clinical Nutrition and Dietetics and Pharmacy [39–50]. See Table 1 for summary of disciplines provided by study. All 12 papers reported intervention from a PT [39–50]. Nine papers described intervention from an OT [39–43, 47–50]. Eight papers described intervention from an SLT [39–41, 44, 46, 47, 49, 50]. Seven papers reported patients received psychological interventions when needed as part of the MDT intervention [40, 41, 43, 45, 46, 49, 50]. In four out of seven studies, this intervention was provided by either a Neuropsychologist [40, 41, 43] or Psychologist [50]. Three of the studies did not report the specific discipline of psychology providing the service [45, 46, 49]. In two studies, Social Workers were part of the MDT [47, 50]. Clinical Nutrition and Dietetics and pharmacy were part of the MDT in only one study [47]. Five out of 12 papers reported input from a physician alongside the rehabilitation programme [40, 41, 44, 47, 50] including a rehabilitation physician, medical doctor, hospitalist or physiatrist and specialists such as geriatricians and liaison psychiatrists.

**Table 1:** Disciplines provided by study

Disciplines	OT	PT	SLT	Dietetics	MSW	Psychology	Pharmacy	Physician
Bellinger	✓	✓	✓					
Di Pietro	✓	✓				✓		
Journey	✓	✓	✓	✓	✓		✓	✓
Piquet	✓	✓	✓		✓	✓		✓
Maltser	✓	✓	✓			✓		✓
Bertolucci		✓	✓					✓
Bompani		✓	✓			✓		
Barbieri		✓	✓			✓		
Cevei	✓	✓						
Coakley	✓	✓						
Chuang	✓	✓	✓			✓		
Cao	✓	✓	✓			✓		✓

Intensity of multidisciplinary rehabilitation was not reported in any study. Eight studies presented detailed information on the nature of rehabilitation intervention [41, 43–46, 48–50]. The description of rehabilitation programmes was heterogenous however domains reported include respiratory/pulmonary rehabilitation, motor and strengthening interventions, training in activities of daily living, energy conservation techniques, advice regarding the home environment and practice of functional mobility and transfers. Please see Table 2 for characteristics of included studies for additional information. The remaining four studies reported only the disciplines that were involved in the MDT intervention or the assessment domains [39, 40, 42, 47].

**Table 2:** Study and patient characteristics

Author/year/ country	Study design	Participants Inclusion/exclusion	Exposure (FITT)	Comparison group	Discharge disposition	Outcome measures	Results
Bellinger et al./2021/ United States of America [39]	Retrospective, descriptive cohort	*Inclusion* - Positive lab test for C19 - admission to inpatient rehabilitation upon discharge from acute care - receipt of a minimum of two out of three therapy disciplines (PT, OT, and ST) *Exclusion* - baseline expressive and/or receptive aphasia, - non-English speakers - readmission to the acute care hospital or leave of absence of more than 3 days from the inpatient rehab - pre-existing illness with a life expectancy of less than 6 months - failure to participate in designated outcome measures (resulting in a lack of data availability) *Participants* - *N* = 35 - Mean age = 68.7 (SD NR) - Sex (female) = 34%	*Frequency* 5 days per week *Intensity* NR *Time* Average 175.64 min per week *Type* 2 out of 3 disciplines (OT, PT, SLT)	None	NR	Completed on admission and discharge from rehabilitation unit 1. IRF PAI: Subsections for self care and mobility 2. 6MWT 3. mBI 4. Orientation Log and Cognitive Log 5. Length of stay (LOS)	1. IRF PAI: Mean difference of 48.2 points, SD not reported between pre and post 2. 6MWT: Mean difference of 472.3 ft between pre and post 3. mBI: Mean difference of 28.95 between pre and post 4. Orientation Log and Cognitive Log: Orientation- mean difference of 8.6 between pre and post Cognitive- mean difference of 4.14 between pre and post 5. Mean LOS = 17.3 days (SD NR) (Mdn = 15 days, range: 5–36 days)
Di Pietro et al./2021/ Italy [43]	Retrospective case report/case series	*Inclusion* - Patients who needed, besides the rehabilitation programme, an extensive neuropsychological evaluation during hospital stay - These patients, aged > 18 years in stabilized respiratory condition (PaO2/FiO2 > 300) - with previous diagnosis of COVID-19 infection proven by a positive PCR nasopharyngeal swab *Exclusion* - patients with delirium - those receiving antipsychotic therapy *Participants* N = 12 Mean age = 64.0 ± 13.7 Median age = 65 (54–73) Sex = NR	*Frequency* Motor rehabilitation = 6 days per week Occupational Therapy = frequency NR Neuropsychology = frequency NR *Intensity* NR *Time* Motor rehabilitation = NR Occupational Therapy = 150 min per week for last 2 weeks of rehabilitation programme Neuropsychology = NR *Type* Motor rehabilitation, Occupational Therapy, Neuropsychological evaluation Exercises focused on muscle strengthening (isotonic and isometric exercises) and conditioning, and bed-to-chair mobility, wheelchair skills, pre-gait (sit to stand), bathroom skills, and activities of daily living (ADL) training	None	NR	1. Cumulative illness rating scale (CIRS 1 = Severity index and CIRS 2 = co-morbidity index) 2. Admission Barthel index 3. Discharge Barthel index 4. Delta BI (difference in BI between admission and discharge) 5. Admission Functional Independence Measure 6. Discharge Functional Independence Measure 7. Delta FIM (difference in FIM between admission and discharge) 8. Mini Mental State Examination 9. Complete neuropsychological assessment battery (point in time) (Forward Digit span Backward Digit span Story test [early recall] Story test [late recall], TMT-a TMT-b FAB Phonemic verbal fluency test Semantic verbal fluency test Rey–Osterrieth complex figure test Clock drawing test) 10. ICU admission (n) and length of stay 11. Normal premorbid state 12. Symptom duration (days)	1. Mean CIRS 1 (severity index) = 2.2 ± 0.5 Mean CIRS 2 (comorbidity index) = 5.6 ± 2.5 2. 41.0 ± 29.5 3. 78.9 ± 16.8 4. 37.9 ± 31.1 5. 70.3 ± 25.1 6. 95.7 ± 26.0 7. 25.4 ± 21.7 8. MMSE: descriptive statistics NR but patients individual scores are reported 9. Neuropsychological assessment battery: Mean Forward Digit span 0–9 = 5.13 ± 0.95 Mean Backward Digit span 0–9 = 3.94 ± 0.57 Mean Story test (early recall), z = − 0.58 ± 1.11 Mean Story test (late recall), z = 0.24 ± 1.60 Mean TMT-a, score = 32.63 ± 22.41 Mean TMT-b, score = 96.17 ± 55.22 Mean FAB, score = 14.58 ± 2.22 Mean Phonemic verbal fluency test = 24.10 ± 6.60 Mean Semantic verbal fluency test = 38.11 ± 6.97 Mean Rey–Osterrieth complex figure test = 30.14 ± 7.81 Mean Clock drawing test = 12.40 ± 2.70 10. ICU Admission N = 6 (50%) ICU stay = mean 26.1 days ± 10.2 11. Normal premorbid state = 10 (83%) 12. Symptom duration (days) Mean = 75.0 ± 42.4
Journeay et al. /2021/ Canada [47]	Retrospective, descriptive cohort	*Inclusion* - Individuals ≥ 18 years - documented COVID-19-positive diagnosis - admitted to a designated COVID-19 inpatient recovery unit *Exclusion* - Rehab stay for longer that 12 weeks - those admitted for palliative care *Participants* N = 41 Median age = 75 (IQR 58- 84) Sex (Male) = 22 (53.7%)	*Frequency* Not reported *Intensity* Not reported *Time* Not reported *Type* Rehabilitative care teams consisted of a hospitalist, physiatrist, nursing, physiotherapist, occupational therapist, speech language pathologist, social worker, recreation therapist, dietitian, pharmacist, ward aides, and environmental services. Specialists available by consultation included geriatrics and consult liaison psychiatry, with internal medicine available	None	Value, n(%) Home = 35 (85.4) Other discharge destinations = NR	1. Single, n (%) 2. Employed, n (%) 3. Admitted from home, n (%) 4. Living alone, n (%) 5. Stairs at home, n (%) 6. Co-morbidities, n (%) Hypertension Diabetes CNS impairment 7. Acute care LOS, Median (IQR) 8. ICU stay, N (%) 9. Rehab LOS (Median, IQR) 10. Ventilator, N (%) 11. Readmissions to acute, n (%) 12. Admission FIM, Median (IQR) 13. Discharge FIM, Median (IQR) 14. Admission MOCA, median (IQR) 15. Admission Diet, n (%) 16. Discharge Diet, n (%) 17. Rehabilitation Client Group, n (%) 18. Affected body functions, n (%)	1. 24 (58.5) 2. 11 (26.8) 3. 37 (90.2) 4. 14 (34.1) 5. 17 (41.5) HTN = 30 (73.2) Diabetes = 15 (36.6) CNS = 15 (36.6) 7. 19 (12–31) 8. 15 (36.6) 9. 16 (13–22) 10. 11 (26.8) 11. 2 (4.9) 12. 85 (75–97) 13. 108.5 (103–118) 14. 25 (20.75- 25) Regular = 35 (85.4) Modified = 6 (14.6) Regular = 39 (95.1) Modified = 2 (4.9) Medically complex = 29 (70.7) Pulmonary disorders = 6 (14.6) Stroke/ortho/debility = 6 (14.6) Neuromusculoskeletal = 30 (73.2) Cardiovascular, haematological, immunological, respiratory = 27 (65.9) Mental function = 12 (29.3) Genitourinary and reproductive = 7 (17.1) Sensory and pain = 4 (9.8) Digestive, metabolic, endocrine = 4 (9.8) Skin and related structures = 3 (7.3) Voice and speech = 0 (0)
Piquet et al./2021/France [50]	Retrospective, descriptive cohort	*Inclusion* - Age 18 or older - The ability and willingness to engage in 2 daily PT sessions 5 days per week *Exclusion* Not reported *Participants* Value, n (%) N = 100 Sex (male) = 66 (66) Median age, IQR = 66 ± 22	*Frequency* Two physical therapy (PT) sessions per day 5 days per week Frequency of OT, SLT and/or Psychology not reported *Intensity* Not reported *Time* Each PT session < 20 min in duration, OT, SLT and/or Psychology session time not reported *Type* Motor strengthening and respiratory rehabilitation Physical education group work 2 occupational therapists, 1 speech therapist, and 1 psychologist also provided service to ward A mobile discharge team comprising a physical medicine and rehabilitation physician, a social worker, and an occupational therapist helped detect and solve any social issues encountered toward returning home. In addition, a dedicated physiotherapist insured proper execution of the self-rehabilitation exercises by video consultation	None	Value, n (%) Home = 75 (75) Relative’s home = 4 (4) COVID-free rehabilitation unit = 15 (15) Acute care = 8 (8)	1. Background and co-morbidities, n (%) 2. Clinical characteristics at time of diagnosis, n (%) 3. Barthel Index 4. time to perform 10 full sit-to-stands as quickly as possible from a standardized 40-cm-height chair, arms folded over the chest, with respiratory rate, oxygen saturation, heart rate, and Borg scale of perceived exertion, recorded before and after 5. Hand grip strength 6. Personal assistance required 7. Deaths, n (%) 8. Intubation, n (%) 9. Nasal O2 at admission to acute, n (%) 10. Nasal O2 at discharge from acute, n (%) 11. Rehab LOS, mean ± SD 12. LOS acute care, mean ± SD 13. Intensive care, n (%)	High blood pressure = 48 (48) Age > 70 = 41 (41) Diabetes = 29 (29) BMI > 30 = 17 (17) Renal failure = 13 (13) Coronaropathy = 1 (1) Stroke = 9 (9) Immunosuppression = 3 (3) Dyspnea = 79 (79) Asthenia = 76 (76) Fever = 73 (73) Cough = 64 (64) Myalgia = 33 (33) Diarrhea = 25 (25) Ageusia = 16 (16) Headache = 14 (14) Anosmia = 13 (13) Pulmonary embolism = 4 (4) Thrombosis = 1 (1) 3. BI: Mean pre infection BI = 94.5 ± 16.2 Mean admission BI = 77.3 ± 26.7 Mean discharge BI = 88.8, ± 24.5 4. Sit to stand frequency increased by 37% Post-sit-to-stand test respiration rate dropped by 9% Borg exertion score after the sit-to-stand test improved by 30% 5. Grip strength among right-handed people (92% of patients) increased by 15% 6. Personal assistance required: Before C19 = 19 (19) After C19 = 24 (24) 7. 2 (2) 8. 13 (13) 9. 77 (77) 10. 58 (58) 11. 9.8 ± 5.1 12. 14.4 ± 8.7 13. 23 (23)
Maltser et al./2021/ United States of America [40]	Retrospective, descriptive cohort	*Inclusion* **Burke** - Demonstrating clinical recovery of symptoms - Have rehabilitation goals - < 6L Supplementary O2 requirements **JFK Johnson** - Must be 7 days from initial symptom onset - At least 3 days since fever resolution - Be without fever reducing meds - Have had an improvement in respiratory symptoms - < or equal 5L supplementary O2 requirements *Participants* **Burke** *N* = 50 Mean = 67.66 ± 12.13 Sex (male) = 29 (58%) **JFK Johnson** *N* = 50 Mean = 64.54 ± 12.16 Sex (male) = 33 (66%)	*Frequency* Daily *Intensity* Not reported *Time* 3 h per day (1 h OT, 1 h SLT, 1 h PT. If no SLT needs, split between PT and OT) *Type* OT SLT PT Also have access to recreation therapy and neuropsychology as needed Information gathered from authors, not reported in research article	Data from the Uniform Data System (UDS) and eRehabData (eRehabData) databases for patients treated for “debility” during the last quarter of 2019 (prepandemic)	**Burke** Home = 31 (62%) Acute hospital = 8 (16%) Subacute rehab = 11 (22%) **JFK Johnson** Home = 46 (92%) Acute hospital = 0 (0) Subacute rehab = 4 (8%)	1. GG Scores related to self care (GG0130) 2. GG scores related to mobility (GG0170) (scales range from 1 to 6, where 1 indicates “dependent” and 6 indicates “independent.”) 3. Rehab LOS, mean ± SD 4. Acute Hospital LOS, mean ± SD 5. Race/ethnicity, n (%)	**Burke** 1. Change in GG score for self care = mean 15.60 ± 5.20 (SD) (P = 0.0001) 2. Change in GG score for mobility = Mean 27.00 ± 6.99 (SD) (P = 0.0001) 3. 15.56 ± 11.91 4. 9.94 ± 10.56 5. White = 25 (50%) Other = 1 (2%) Asian = 2 (4%) Black = 13 (26%) Hispanic = 9 (18%) Unknown = 0 (0%) **JFK Johnson** 1. Change in GG score for self care = mean 14.04 ± 6.93 (SD) P < 0.0001 2. Change in GG score for mobility = mean 32.68 ± 13.52 (SD) P < 0.0001 3. 15.72 ± 6.65 4. 29.42 ± 23.45 5. White = 14 (28%) Other = 1 (2%) Asian = 9 (18%) Black = 15 (30%) Hispanic = 11 (22%) Unknown = 0 (0%)
Bertolucci et al./2021/Italy [44]	Prospective, descriptive cohort	All consecutive patients requiring rehabilitative programme due to complex disabilities following COVID-19 pneumoniae referred to the Rehabilitation Unit of Versilia Hospital in Italy between March 30 and August 10, 2020 were enrolled *Inclusion* - severe respiratory failure which required hospitalization in Intensive Care Unit or Medical ward requiring noninvasive or invasive ventilation in acute phase - hemodynamics and respiratory stability at admission, without catecholamine infusion or ventilation, even if patients needed the delivery of high oxygen flow with FiO2 up to 60%; - respiratory trend towards improvement; - sufficient autonomy in ADL before infection testified by anamnestic Barthel Index (BI) > = 50 - presence of actual severe disability - absence of fever in the previous 48 h - current or past laboratory-confirmed SARS-CoV-2 infection *Exclusion* Not reported *Participants* N = 39 Mean age = 67.8 ± 10.8 Sex (female) = 15 (38.46%)	*Frequency* Daily therapy sessions as patients were able *Intensity* *Time* 2 h of rehabilitation per day, as patients were able *Type* pulmonary rehabilitation: - training for breath control by abdominal diaphragmatic direct ventilation, chest expansion, controlled breathing, diaphragmatic re-education, volume increasing - airways cleaning by bronchus suction and airways unblocking, use of Positive Expiratory Pressure (PEP) devices motor rehabilitation: - active-assisted and active joint mobilization of the 4 limbs, also with mechanical devices - muscle strengthening - active postural changes, readjustment of postural reflexes, coordination exercises for trunk control - recovery of standing position - reconditioning of walking and effort by interval training and continuous training in order to increase the endurance and prescription of orthosis Swallowing rehabilitation: - sensory-motor stimulation - postural compensation - change in food consistency - progressive introduction of foods of different consistency - oral hygiene	None	Value, n (%) Home = 38 (97.44) Acute hospital = 1 (2.56%)	1. Cummulative Illness Rating Scale for medical comorbidity and severity 2. Presence of obesity and diabetes 3. Virological data and clinical course 4. Clinical features at admission 5. Clinical features at discharge 6. Functional measures (BI and FAC) 7. Rehabilitation LOS 8. ICU/Acute ward LOS 9. ICU admission, n (%)	1. Median index of CIRS comorbidity = 1 (which means 1 body apparatus/systems affected by disease which requires therapy) Median index of CIRS severity = 1.15 2. Obesity = 14 (35.8%) Diabetes = 10 (25.6%) No comorbidity = 14 (CIRS score, 0) 3. Fifteen out of 39 subjects had nasal/throat swabs positivity for SARS-CoV-2 at admission to rehabilitation 14 had viral clearance by 2 negative nasal/ throat swabs in the previous 48 h Re-positive swabs after viral clearance was detected in 17 patients 2 patients were discharged still positive whilst the others showed two negative swabs at discharge Admitted from ICU = 32 (82.05%) Admitted from medical wards = 7 (17.95%) orotracheal intubation = 28 (71.8%) Duration of intubation = range of 4–36 days Prone ventilation = 17 (43.6%) Bacterial superinfection at admission = 23 (58.9) 4. 8 out of 39 patients had no oxygen supplementation at admission Admission mean PaO2/FiO2 = 360,7 ± 122,9 Tracheostomy at admission = 11 (28.21%) Dysphagia and fed via Nasogastric tube = 7 (17.95%) Peripheral nervous system impairment = 7 (17.95%) Rectal colonisation = 28 (71.8%) Corticosteroid use = 19 (48.7%) Mental confusion—= 17.9% Antipsychotic drugs = 11 (28.2%) 5. Without oxygen supplementation at discharge = 31 (79.4%) Tracheostomy removal = 38 (97.44%) Complete oral alimentation = 39 (100%) Mental confusion = 0 (0%) Corticosteroid use = 4 (10.2%) Antipsychotics = 5 (12.8%) Rectal colonisation = 28 (71.8%) Anamnestic BI = Median score of 5 (5-5) Admission BI = Median score of 7.5 (0- 10) Discharge BI = Median score of 65 (60- 85) Anamnestic FAC = Median score of 100 (100- 100) Admission FAC = Median score of 0 (0–0) Discharge FAC = Median score of 3 (3–4) 7. Mean rehab LOS = 25.5 ± 16.3 8. Mean ICU or acute ward LOS = 46.4 ± 20.9 9. 32 (82%)
Bompani et al./2023/Switzerland [46]	Retrospective, pre-post intervention cohort study	*Inclusion* For patients with a severe acute respiratory syndrome coronavirus 2 (SARS-CoV-2)-positive nasopharyngeal swab: 1. a recent chest computed tomography scan or X-ray with evidence of significant improvement versus baseline 2. arterial oxygen partial pressure (PaO2)/fractional inspired oxygen (FiO2) ratio (P/F ratio) > 300 with FiO2 35% during recovery in the ICU 3. Apyretic for at least 3 days; 4. 90 mmHg < systolic blood pressure < diastolic blood pressure < 90 mmHg For patients with a negative nasopharyngeal swab for SARS CoV-2: 1. apyretic for at least 3 days, and 2. at least two consecutive negative swabs with an interval of at least 48 h between swabs *Exclusion* - Patients who were under psychotropic drugs prior to study inclusion - those with COVID-19 encephalitis - patients with signs of dementia - patients with pre-COVID 19 history of neurological or psychiatric diagnosis *Participants* N = 66 Mean age = 70.14 ± 10.82 Sex (male) = 39 (59%)	*Frequency* - Respiratory and neuromotor domains: Daily - Psychological intervention: Dependant on patients needs - Speech and Nutritional interventions: daily for those who were mechanically ventilated *Intensity* NR *Time* - Respiratory domain: 30–40 min according to patient’s tolerance - Neuromotor domain: 30 min - Psychology: NR - Speech and nutrition: 30–45 min according to patients tolerance *Type* Respiratory domain: respiratory exercises, such as deep, slow breathing, and chest expansion combined with shoulder expansion in order to reduce the spread of droplets. Once negative for SARS-CoV-2, aerosol therapy could be introduced and active breathing, as well as training with positive expiratory pressure, were started *Neuromotor domain:* Aimed at preservation of joint mobility and prevention of muscle wasting *Psychology:* Aimed at addressing emotional and traumatic issues *Speech and nutrition:* Aimed to improve speech and swallow skills which may have been compromised due to mechanical ventilation	None	NR	1. FIM at admission (TO) and discharge (T1) from rehabilitation. Reported as mean, ± SD and range. Motor, cognitive and total scores were calculated On admission to rehabilitation only: 2. Cumulative illness rating scale 3. BMI 4. Nutritional Risk Screening-2002 (NRS-2002) system 5. MMSE 6. Digit Span Forward task 7. Story-Recall test 8. Frontal Assessment Battery 9. Digit Span Backward task 10. HADS 11. Chalder Fatigue Scale On discharge only: 12. Rehabilitation Effectiveness index (REs) 13. Rehabilitation LOS (Days) 14. ICU admission and mechanical ventilation, n (%)	T0 total FIM score: 55.42 ± 25.97 (18- 116) T1 total FIM score: 93.82 ± 20.83 (38- 125) T0 FIM cognitive score: 21.37 ± 8.01 (5- 33) T1 FIM cognitive score: 27.55 ± 5.52 (10- 35) T0 FIM motor score: 34.34 ± 19.84 (13- 85) T1 FIM motor score: 66.27 ± 16.45 (27- 90) CIRS severity index (0–56): 1.76 ± 0.57 (0–3) CIRS comorbidity index (0–12): 7.68 ± 2.35 (3–11) CIRS Psychiatric index (0–4): 1.42 ± 1.84 (0–4) 3. 28.92 ± 6.91 (20–51) 4. 4.58 ± 1.03 (3–7) 5. 25.02 ± 5.84 (3–30) 6. 5.25 ± 1.23 (3–9) 7. 12 ± 5.76 (0–24) 8. 13.3 ± 1.23 (2–7) 9. 3.7 ± 1.23 (2–7) 10. HADs anxiety: 5.26 ± 4.18 (0–17) HADs depression: 4.57 ± 3.49 (0–17) 11 6.29 ± 2.3 (0–13) 12. 51.88 ± 25.75 (4–94) 13. 41.83 ± 25.29 (8–146) 14. 45 (68.18%)
Barbieri et al. /2022/ Switzerland [45]	Quasi experimental	*Inclusion* Patients admitted to hospital with severe coronaravirus disease For patients with a SARS-CoV-2-positive nasopharyngeal swab: - a recent chest computed tomography or X-ray with evidence of significant improvement versus baseline - arterial oxygen partial pressure (PaO2)/fractional inspired oxygen (FiO2) ratio > 300 with FiO2 35%; - apyretic for 3 days - systolic blood pressure < 140 mmHg and diastolic blood pressure < 90 mmHg For patients with a negative nasopharyngeal swab for SARS-CoV-2: - apyretic for 3 days - at least two consecutive negative swabs with at least a 48-h interval between swabs *Exclusion* - Patients under existing prescription for psychotropic drugs - those with COVID-19 encephalitis - or with signs of dementia *Participants* N = 53 Mean age = 67.9 ± 8.73 Age range = 49–92 Sex (male) = 37 (69.8%)	*Frequency* Respiratory and neuromotor domains: daily Psychological intervention: dependent on patient need *Intensity* NR *Time* - Respiratory: 30–45 min depending on tolerance - Neuromotor: 30 min - Psychological: NR *Type* *Respiratory:* aimed at reducing breathing difficulties and perception of dyspnoea, and reducing incidence of complications Patients who remained positive for SARS-CoV-2 underwent a rehabilitative protocol that included respiratory exercises such as deep, slow breathing, and chest expansion combined with shoulder expansion in order to reduce the spread of droplets. Breathing exercise helped patients to fully re-expand the lungs and to further the progression of airway secretions from small to large airway, thus reducing alveolar dead space Once negative for SARS-CoV-2, aerosol therapy was introduced and active breathing, as well as training with positive expiratory pressure, were started Neuromotor: programme to preserve joint mobility and to prevent muscle wasting Psychological: aimed to address the emotional and traumatic issues related to the disease itself and to the prolonged isolation of hospitalization	None	NR	1. FIM at admission (T0) and discharge (T1) from rehabilitation. Reported as mean, ± SD and range. Motor, cognitive and total scores were calculated On admission to rehabilitation only: 2. Cumulative illness rating scale 3. BMI 4. Nutritional Risk Screening-2002 (NRS-2002) system 5. 30 s sit to stand test (number of repetitions) 6. Jamar hand dynamometer (mean of right and left as kg) 7. Perceived pain by VAS On discharge only: 8. Rehabilitation Effectiveness index (REs) 9. Rehabilitation LOS (days)	T0 total FIM score: 74.52 ± 24.28 (21–123) T1 total FIM score: 107.16 ± 21.7 (21–126) T0 FIM cognitive score: 28.62 ± 6.62 (8–35) T1 FIM cognitive score: 30.86 ± 5.68 (8–35) T0 FIM motor score: 45.9 ± 19.75 (13–88) T1 FIM motor score: 76.3 ± 16.84 (13–91) CIRS severity index (0–56): 1.51 ± 0.48 (0.61–2.61) CIRS comorbidity index (0–12): 6.69 ± 2.39 (2–12) 3. 28.92 ± 6.53 (19–54) 4. 3.92 ± 1.35 (2–6) 5. 3.72 ± 3.56 (0–11) 6. 18.82 ± 8.96 (2–41) 7. 2.0 ± 2.47 (0–8) 8. 68 ± 26.06 (0–100) 9. 31.81 ± 20.37 (9–136)
Cevei et al., 2022 Romania [48]	Case series	*Inclusion* Patients admitted to acute hospital for severe coronavirus illness requiring rehabilitation - Patients > 80 years old, previously diagnosed with severe SARS-CoV-2 infection - with no clinical and biological signs of acute viral disease, - with loss of autonomy for activities of daily living 1 month after the diagnosis - musculoskeletal dysfunction - inability to walk *Exclusion* - Patients with dyspnea at rest - O2 saturation under 93% - cardiorespiratory instability *Participants* N = 6 Mean age ± SD = 85.33 ± 3.07 Sex (male) = 4 (67%)	*Frequency* Occupational therapy and Physical therapy twice daily Robotic-assisted gait training and massage therapy daily *Intensity* NR *Time* Physical therapy and Occupational therapy 30 min 2times/day Robotic assisted gait training 15–30 min per day Massage therapy 20 min per day *Type* Physical therapy Sessions focused on passive and active joint range of movement, strengthening exercises, transfers re-education, and co-ordination and balance re-education Occupational Therapy Sessions focused on restoration of active mobility, strength, and coordination in the upper and lower body, acquisition of maximum degree of functional independence in self-care, establishing balance between rest, occupational, and recreational activities, and to improve ADLs and to increase the quality of life by optimizing the patient’s home environment to his/her individual abilities Robotic assisted gait training Sessions involved repetitive movements associated with visual, auditory, and tactile feedback Massage therapy For the upper body sessions aimed to improve muscle relaxation, reduce the severity of muscle soreness, soften tender and trigger points, and to have a general sedative effect. For the lower body sessions targeted circulation improvement, facilitating an increase of mobility of the joints and soft tissues, and reducing edema	None	NR	Gathered at admission (T0) and discharge (T1) from rehabilitation, reported as mean ± SD 1. BI 2. FIM 3. Grip strength 4. CIRS-G At discharge 5. LOS in rehabilitation hospital *Hip flexion and manual muscle testing were also reported for the cohort but not reported here. Please see original paper for details*	T0 BI: 18.33 ± 23.59 T1 BI: 50.83 ± 36.39 T0 FIM: 50.67 ± 31.57 T1 FIM: 75.00 ± 31.16 T0 Right 12.72 ± 3.81 T0 Left 13.61 ± 5.93 T1 Right 18.44 ± 3.38 T1 Left 17.56 ± 5.62 4. 16.33 ± 8.68 5. 17 ± 3.79
Coakley et al./2023/USA [42]	Retrospective descriptive cohort study	*Inclusion* All adult patients (ages 18 +) admitted to acute hospital who tested positive for COVID-19 were included in the study. This included patients who did and did not receive rehabilitation and those who did and did not require ICU admission. Data were extracted for those who received rehabilitation from the main sample for purpose of this review *Exclusion* NR *Participants* N = 54 Mean age = 68 ± 16 Sex (male) = 26 (48%)	*Frequency* Daily *Intensity* NR *Time* 3 or more hours per day *Type* Rehabilitation programme consisted of Occupational Therapy and Physical Therapy. Nature of interventions were not described but assessment domains were described in detail This included: - proximal strength - distal strength - cognition - sitting and standing balance - sensation of upper and lower extremities - proprioception of upper and lower extremities - coordination of upper and lower extremities - activity tolerance - Functional assessment of bed mobility, activities of daily living, and ambulation	1. No Therapy, No ICU group 2. No Therapy, ICU group 3. Therapy, ICU group	Reported as n (%) Home = 36 (68) Long term care facility = 3 (6) Subacute rehabilitation = 14 (26) Other = 0 (0)	1. The Boston AM-PAC “6 Clicks” Basic Mobility Inpatient Short Form (reported as mean ± SD) 2. The Boston AM-PAC “6 Clicks” Daily Activity Inpatient Short Form 3. Co-morbidities, n (%) 4. Hospital LOS, median (IQR) 5. Mortality, n (%) 6. ICU, N (%)	Pre: 17.1 ± 4.3 Post: 17.9 ± 4.1 Mean difference: 1.0 ± 2.3 Pre: 17.1 ± 3.9 Post: 16.7 ± 3.7 Mean difference: 0.7 ± 2.0 Chronic lung disease: 13 (25) Diabetes: 28 (53) Cardiovascular disease: 46 (87) Renal disease: 12 (23) Liver disease: 5 (9) Immunosuppressive co-morbidity: 4 (8) Neurological co-morbidity: 10 (19) Cancer: 8 (15) Smoker: 4 (8) 4. 6 (3- 9) 5. 1 (2) 6. 0 (0)
Chuang et al./2022/Taiwan [49]	Case series	*Inclusion* - Two consecutive sets PCR test with negative results or a cycle threshold value exceeding 34 within 7 days - No oxygen requirement greater than 3 L per minute - Stable vital signs including body temperature, blood pressure, and heart rate - Need for multidisciplinary rehabilitation - Clear consciousness and able to follow up simple orders Exclusion NR *Participants* N = 5 Mean age = 73.4 Sex (male) = 4 (80%)	*Frequency* 5 days/week for PT, OT, SLT As indicated for Psychology *Intensity* NR *Time* Minimum 20 min for each discipline, as tolerated *Type* PT Motor strengthening, balance training, aerobic training and ambulation training according to patient ability. Outside treatment sessions, patients were instructed in individualized, low intensity and multiple repetition exercises with the aid of videos or pictures. Breathing exercises to relieve exertional dyspnea and control inspiratory/ expiratory rhythm OT Therapy to address basic activities of living, energy conservation, evaluation of adaptive devices and environmental adaptations required for discharge SLT Swallow assessment and speech assessment Psychology NR	None	Home = 4 (80%) Nursing home = 1 (20%)	Gathered at admission (T0) and discharge (T1) from rehabilitation: 1. BI 2. FAC 3. FOIS 4. BMI Raw data for the above measures were extracted from the case series and the author calculated mean and SD values At discharge 5. ICU LOS, Median 6. ICU, N (%) 7. Rehab LOS 8. Need for feeding tube, N (%)	T0 BI: 26 ± 23.82 T1 BI: 71 ± 20.43 T0 FAC: 1.6 ± 1.14 T1 FAC: 3.4 ± 0.89 T0 FOIS: 4.2 ± 2.95 T1 FOIS: 6.4 ± 0.55 T0 BMI: 22.3 ± 6.06 T1 BMI: 19.56 ± 6.32 5. 17 6. 5 (100) 7. Median = 17 (mean 22.2 ± 13.74 as calculated by this author as raw data was available) 8. 2 (20%)
Cao et al./2022/USA [41]	Retrospective cohort study	*Inclusion* Any patient within the hospital or outside the hospital who met the following admission criteria was considered for admission to the rehabilitation unit: - Seven days from diagnosis of COVID19 - at least 72 h non-febrile without taking fever reducing medication - may have a tracheostomy but no need for prescribed suction - oxygen need < 5 L at rest - improving Covid19-related symptoms and in need of rehabilitation, while also considering individual psychosocial needs such as home environment and impact on family members - ability to tolerate and participate three hours per day of therapy, 5–7 days per week *Exclusion* None *Participants* N = 59 Mean age (SD) = 65 ± 13.2 Sex (male) = 31 (52.5%)	*Frequency* 5- 7 days per week *Intensity* NR *Time* 3 h per day *Type* Each patient was assessed on admission by each member of the MDT; physiatrists and medical consultants, physical therapists, occupational therapists, speech therapists, neuropsychologists, respiratory therapists, and rehabilitation nursing The MDT programme involved: pulmonary rehabilitation including: - optimization of overall medical management - progressive exercise protocol with closely monitored vital signs and pulse oximetry - energy conservation techniques - respiratory physiotherapy - Mobility and daily activity functional training activities tailored to address the individual’s functional deficits - For patients with cognitive impairment, cognitive therapy involved a combination of remediation through direct training, metacognitive strategy instruction and use of compensatory techniques - All patients were able to access daily speech/swallow pathology and neuropsychology service for cognition assessment and psychological support as well, if needed	None	Group 1: Patients had admission to ICU Home = 11 (78.6%) Skilled nursing facility = 1 (7.1%) Acute hospital = 2 (14.3%) Group 2: Patients did not have ICU admission Home = 44 (97.8%) Skilled nursing facility = 0 (0%) Acute hospital = 1 (2.2%)	1. Co-morbidities, n (%) 2. Complications 3. Need for invasive mechanical ventilation in ICU, n (%) For the following outcomes, the cohort is reported according to ICU admission status: 4. Admission and discharge scores for GG Self-Care of the Centers for Medicare & Medicaid Services issued IRF-PAI Version 3.0. (reported as mean ± SD) 5. Pre and post GG Mobility Item of the Centres for Medicare and Medicaid Services issued by IRF-PAI Version 3.0. (reported as mean ± SD) 6. BMI on transfer to IRF (reported as mean ± SD) 7. LOS, Median (IQR) a) ICU b) Acute care c) Rehabilitation unit 8. Presence of dysphagia on admission, n (%) 9. Presence of dysphagia on discharge, n (%) 10. Oxygen requirement at admission to rehab, n (%) 11. Oxygen requirement on discharge from rehab, n (%) 12. Discharge disposition, n (%)	Hypertension: 48 (81.4%) Type II Diabetes: 23 (40%) Cardiac dysfunction: 23 (40%) COPD: 8 (4%) Kidney disease: 13 (22%) Malignance: 5 (8.5%) DVT: 5 (8.5%) Pulmonary embolism 5 (8.5%) 3. 14 (23.7%) Group 1 Admission: 19 ± 19.2 Discharge: 35 ± 8.3 Change: 17 ± 7.5 Group 2 Admission: 20 ± 5.4 Discharge: 34 ± 9.2 Change: 14 ± 6.4 Group 1 Admission: 27 ± 8.0 Discharge: 71 ± 22.3 Change: 44 ± 21.3 Group 2 Admission: 31 ± 12.4 Discharge: 72 ± 22.96 Change: 41 ± 18.2 Those ventilated: 30 ± 6.4 No ventilation 30 ± 7.6 Group 1 a) 9.0 (4.0- 11.8) b) 18 (16- 26) c) 13 (10, 16) Group 2 a) N/A b) 10 (7- 13) c) 12.5 (11- 15.3) Group 1 5 (35.7%) Group 2 7 (15.6%) Group 1 0 (0%) Group 2 1 (2%) Group 1 6 (42.9%) Group 2 21 (46.7%) Group 1 0 (0%) Group 2 2 (4.4%)

*NR* Not reported, *LOS* Length of stay, *ICU* Intensive Care Unit, *IQR* Interquartile range, *OT* Occupational Therapy, *PT* Physio/Physical therapy, *SLT* Speech and Language Therapy, *BI* Barthel Index, *FAC* Functional Ambulation Category, *IRF* Inpatient Rehabilitation Facility, *IRF PAI* Inpatient Rehabilitation Facility Patient Assessment Instrument, *6MWT* Six Minute Walk Test, *m BI* Modified Barthel Index, *MMSE* Mini Mental State Examination, *HADS* Hospital Anxiety and Depression Scale, *CIRS* Cumulative Illness Rating Scale, *CIRS-G* Cumulative illness Rating Scale Geriatric, *VAS* Visual analogue scale, *FOIS* functional oral intake scale

### Methodological quality

Table 3 details results of the CASP checklist for cohort studies and Table 4 details results of the JBI critical appraisal tool applied to the case studies included in this systematic review.

**Table 3:** CASP checklist

	**CASP item**	**Bellinger**	**Journeay**	**Piquet**	**Maltser**	**Bertolucci**	**Bompani**	**Barbieri**	**Coakley**	**Cao**
1	Clearly focused issue/question?	Yes	Yes	Yes	Yes	Yes	Yes	Yes	Yes	Yes
2	Was the cohort recruited in an acceptable way?	No	No	No	No	No	No	No	No	No
3	Was the exposure accurately measured to minimise bias?	Yes	No	Yes	Yes	Yes	Yes	Yes	Yes	Yes
4	Was the outcome accurately measured to minimise bias?	Yes	Yes	Yes	Yes	Yes	Yes	Yes	Yes	Yes
5a	Have the authors identified all important confounding factors?	No	No	Yes	No	No	Yes	Yes	No	Yes
5b	Have they taken account of the confounding factors in the design and/or analysis?	No	No	Yes	No	No	Yes	Yes	Yes	Yes
6a	Was the follow up of subjects complete enough?	No	Yes	Yes	Yes	Yes	Yes	Yes	Yes	Yes
6b	Was the follow up of subjects long enough?	No	No	No	No	No	Yes	No	No	No
9	Do you believe the results?	Yes	Yes	Yes	Yes	Yes	Yes	Yes	Yes	Yes
10	Can the results be applied to the local population?	Yes	Yes	Yes	Yes	Yes	Yes	Yes	Yes	Yes
11	Do the results of this study fit with other available evidence?	Yes	Yes	Yes	Yes	Yes	Yes	Yes	Yes	Yes

**Table 4:** JBI critical appraisal tool

	**Question**	**Di Pietro**	**Cevei**	**Chuang**
1	Were there clear criteria for inclusion in the case series?	Yes	Yes	Yes
2	Was the condition measured in a standard, reliable way for all participants included in the case series?	Yes	Yes	Yes
3	Were valid methods used for identification of the condition for all participants included in the case series?	Yes	Yes	Yes
4	Did the case series have consecutive inclusion of participants?	Yes	Yes	Unclear
5	Did the case series have complete inclusion of participants?	Yes	Yes	Unclear
6	Was there clear reporting of the demographics of the participants in the study?	Yes	Yes	Yes
7	Was there clear reporting of clinical information of the participants?	Yes	Yes	Yes
8	Were the outcomes or follow-up results of cases clearly reported?	Yes	Yes	Yes
9	Was there clear reporting of the presenting sites’/clinics’ demographic information	Yes	Yes	Yes
10	Was the statistical analysis appropriate?	Yes	Yes	Yes

#### Cohort studies

All cohort studies in the review addressed a clearly focused question [39–42, 44–47, 50]. In eight out of nine studies, the exposure was accurately measured to minimise bias [39–42, 44–46, 50]. In eight out of nine studies the follow up of patients was deemed adequate [40–42, 44–47, 50]. However, all nine studies recruited a convenience sample of patients. Four of the studies identified all important confounding factors for results[41, 45, 46, 50] and five studies took these factors into account when designing the methods or completing analysis [41, 42, 45, 46, 50]. In addition, no study followed up patients for long enough evidenced by the absence of follow up beyond the point of discharge.

#### Case study/Case report

There were two case report/case series of high quality in this systematic review satisfactorily meeting all criteria in the JBI checklist(43, 48). The complete and consecutive inclusion of participants by Chuang and colleagues was unclear however it met all other quality criteria in the JBI checklist(49). Of relevance to our secondary outcomes, Di Pietro and colleagues documented an intent to follow up patients at eight to 10 months [43] however the results of this review have not been published to the authors knowledge. Table 4 details results of the JBI critical appraisal tool.

Table 5 details results of GRADE analysis for the primary outcome of functional ability. Analysis discovered very low certainty for quality across studies meaning the true effect is probably markedly different from the estimated effect.

**Table 5:** GRADE assessment of outcome: functional ability

**Multidisciplinary rehabilitation for older adults with COVID-19** **Patient/population**: older adults with COVID-19 **Setting**: Acute or post-acute hospital setting **Intervention**: Multidisciplinary rehabilitation **Comparison**: None							
Study Design	Measurement Instrument	Risk of Bias	Inconsistency	Indirectness	Imprecision	Estimate of Effect [95% CI]	Quality
- Retrospective descriptive cohort (*N* = 6) -Retrospective case series (*N* = 1) -Prospective descriptive cohort (*N* = 1) -Retrospective pre-post intervention cohort (*N* = 1) -Quasi-experimental (*N* = 1) -Case series (*N* = 2)	-Barthel Index (*N* = 5) -Modified Barthel Index (*N* = 1) -Functional Independence Measure (*N* = 3) -Boston AM-PAC “6 Clicks” Daily Activity Short Form (*N* = 1) -US Centres for Medicare and Medicaid Services mandated section GG Functional Abilities Score (*N* = 1)	Serious^a^	Very Serious (*I*^*2*^ = 91%)	Serious	Not Serious	1.46 [0.94, 1.98]	Very Low Certainty

^a^Nine studies recruited a convenience sample, eight studies did not follow up patients for long enough, 4 studies did not account for confounding factors

### Primary outcome

#### Functional ability

**Fig. 2:**
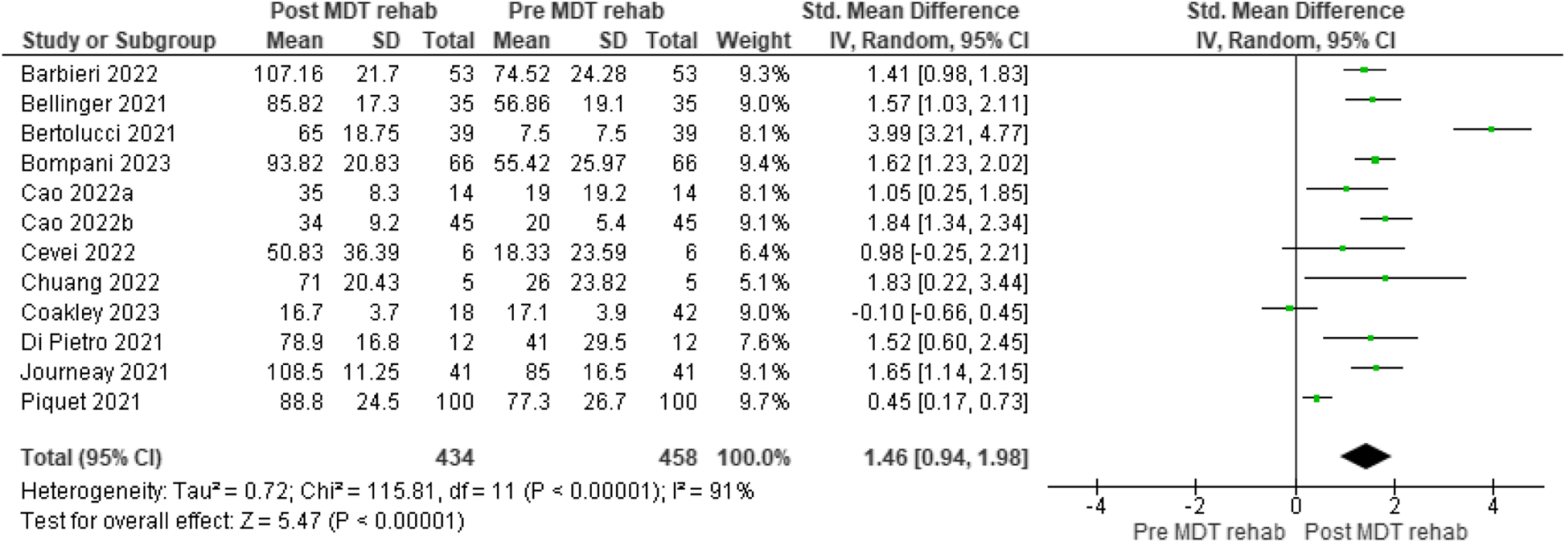
Functional ability pre and post MDT rehabilitation in the acute setting

Functional ability was assessed pre and post MDT intervention in all studies. The validated measures used in eleven of the 12 studies for meta-analysis were the Barthel Index (BI) [43, 44, 48–50], the Modified Barthel Index (m BI) [39], the Functional Independence Measure (FIM) [45–47], the Boston AM-PAC “6 Clicks” Daily Activity Inpatient Short Form [42] and the US Centres for Medicare and Medicaid Services mandated section GG Functional Abilities score [41]. Figure 2 demonstrates that there was a statistically significant improvement in functional ability among older adults with COVID-19 who received multidisciplinary rehabilitation (REM, SMD = 1.46, 95% CI 0.94 to 1.98). Heterogeneity across the studies was significant and considerable (*p* < 0.00001, I^2^ = 91%). However, random effects meta-regression showed age (*p* = 0.747), gender—% males (*p* = 0.314), and number of disciplines (*p* = 0.784) did not moderate functional outcome post-MDT or explain sources of heterogeneity. See Table 6 for results of meta-regression. In the study by Maltser et al., authors reported a statistically significant change in functional ability measures following their described rehabilitation protocol [40]. This change was measured using the US Centres for Medicare and Medicaid Services mandated section GG Functional Abilities and Goals of the Improving Post-Acute Care Transformation Act. GG scores measure changes related to self-care (GG0130) and mobility (GG0170).

**Table 6:** Random Effects Meta-Regression

Moderator	k	Coefficient	SE	Z value	p	LL	UL
Age	10	-0.021	0.065	-0.323	0.747	-0.148	0.106
Gender (% male)	12	0.038	0.038	1.008	0.314	-0.036	0.113
No. Disciplines	12	0.058	0.211	0.274	0.784	-0.356	0.471
LOS	10	0.028	0.027	1.039	0.299	-0.025	0.080

*k* Number of samples, *SE* Standard error, *p* Significance value of named predictor, *LL* Lower limit, *UL* Upper limit

### Secondary outcomes

#### Rehabilitation length of stay

**Fig. 3:**
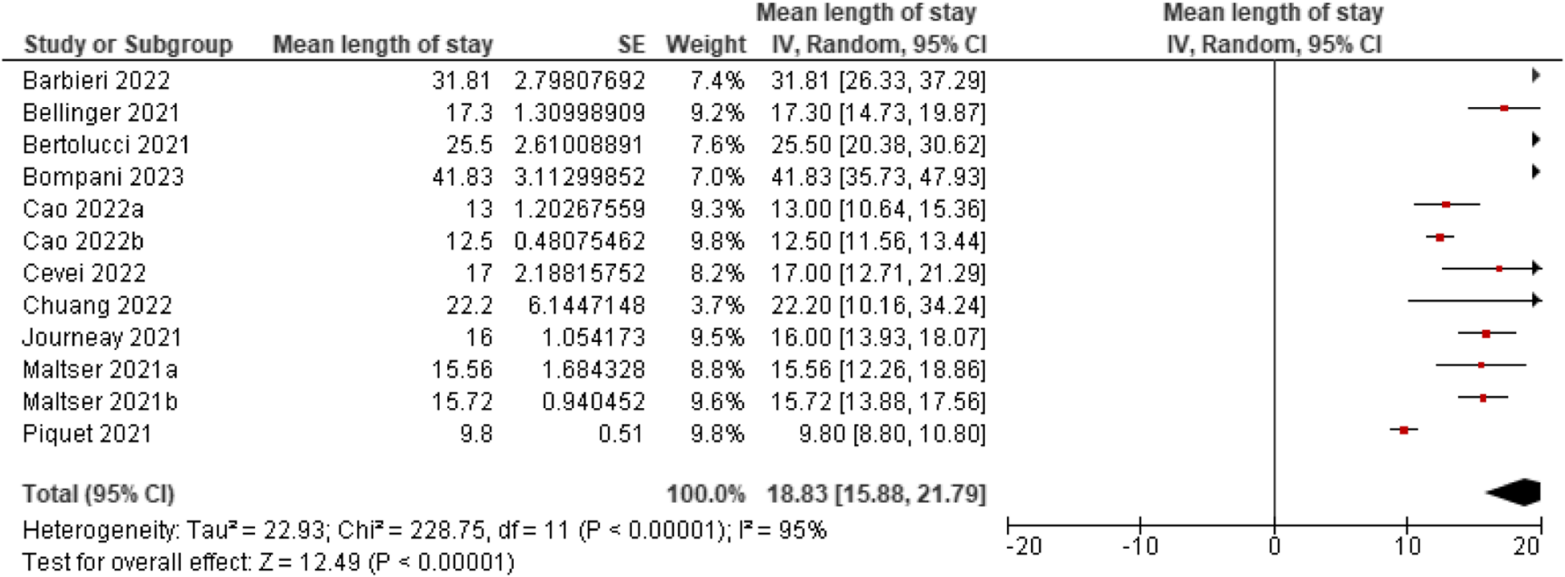
Rehabilitation length of stay among older adults with COVID-19

Rehabilitation length of stay was measured across 12 studies. The mean length of stay for older adults in rehabilitation units was 19 days (95%CI, 15.88–21.79 days). Heterogeneity was substantial across the pooled studies (*p* < 0.00001, I^2^ = 95%). See Fig. 3. Data from 10 of these studies could be pooled to examine the moderating effect of rehab length of stay on functional outcomes. Meta-regression showed length of stay did not significantly predict functional outcome post-MDT, *(p* = 0.299).

#### Acute hospital length of stay

**Fig. 4:**
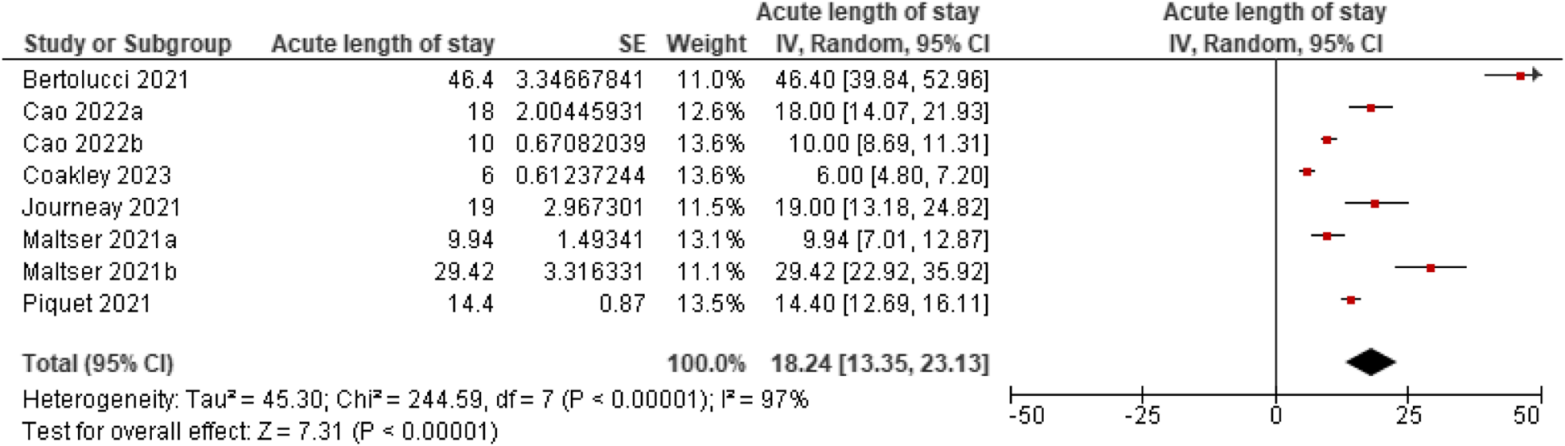
Acute hospital length of stay among older adults with COVID-19

Acute hospital length of stay was measured across six studies comprising eight cohorts. The mean acute hospital length of stay for older adults was 18 days (95%CI, 13.35- 23.13 days). Heterogeneity was significant (*p* < 0.00001, I^2^ = 97%). See Fig. 4. Insufficient number of studies were available to analyse acute hospital length of stay as a moderator on functional outcomes post-MDT.

#### Discharge disposition

Seven studies reported discharge disposition of older adults. The proportion of older adults who were discharged directly home from the acute setting ranged from 62 to 97% [40–42, 44, 47, 49, 50]. Other discharge destinations included a relative’s home, COVID-19 free rehabilitation unit, sub-acute rehabilitation units, skilled nursing facilities and return to acute care.

#### Mortality

Two studies reported 2% mortality of older persons [42, 50], during rehabilitative care. Piquet and colleagues’ patient cohort had a mean length of stay in the acute hospital of 14.4 days and 9.8 days in rehabilitation and 23% required intensive care unit care [50]. Coakley and colleagues had a median length of stay of 6 days in the acute setting with 0% admission to ICU [42].

#### Primary/Community and secondary healthcare utilisation

No studies reported primary and secondary healthcare utilisation, including unplanned Emergency Department return, or unscheduled hospital admission after discharge from rehabilitation units.

None of the studies reported on long-term effects of COVID-19 at discharge from rehabilitation units or at agreed follow-up points in time. Some studies did describe patients need for supplementary oxygen [41, 44, 50] on discharge, reporting prevalence of 4 and 58%. In addition, Bertolucci also reported persisting symptoms at the time of discharge. The author reports that tracheostomies were removed in 97.44% of patients on discharge from rehabilitation (28.22% of patients had a tracheostomy on admission), 100% of patients achieved complete oral alimentation, zero patients presented with mental confusion, 10.2% of patients were continuing to be prescribed corticosteroids and 12.8% were continuing to be prescribed antipsychotics.

## Discussion

This review aimed to describe the clinical characteristics, functional and process outcomes of older adults with COVID-19 who received MDT rehabilitation in the inpatient acute or post-acute hospital setting. There was heterogeneity across the 12 included studies with regards to study design, MDT intervention provided, and outcomes measured. There was a significant improvement in functional ability among older adults with COVID-19 who received MDT rehabilitation, but only two studies had a comparator group [40, 42]. The proportion of older adults who were discharged directly home from the acute setting ranged from 62 to 97%. No studies followed up patients after discharge or reported on long term effects of COVID-19 on discharge from rehabilitation units.

The key finding of our review is that MDT rehabilitation for older adults with COVID-19 in acute or post-acute inpatient hospital setting resulted in statistically significant improvement in function. Moreover, this improvement in functioning was not moderated by length of rehabilitation stay. Our primary outcome, function, aligns with the WHO agenda for healthy ageing globally [51] which recognises society’s duty to facilitate the rights of the older adult to healthy ageing. Our findings support guidelines by the European Geriatric Medicine Society (EuGMS) [28] and the WHO [21] which recommend MDT rehabilitation for older adults hospitalised with COVID-19.

This review found that older adults stayed in hospital for an average of 18 days (95%CI, 13.35- 23.13 days) and in rehabilitation units for 19 days (95%CI, 15.88–21.79 days). Mortality was not routinely reported across studies, but the incidence was low (2%). Rehabilitation length of stay following COVID-19 has already been reported in the literature however in a younger cohort of patients, where length of stays ranged from 11 days to 44.96 days [30, 52–59]. Most of this evidence represents patients of high illness acuity with patients described as having critical illness or severe illness or requiring intensive care unit treatment [30, 52, 55, 57–59]. This is comparable to the evidence presented in this review, where older adults required ICU admission in seven out of 12 studies [41, 43, 44, 46, 47, 49, 50]. In a study by O’Kelly and colleagues, authors reported patients had a median length of stay of 9 days, with 17% requiring ICU admission, however again patients were younger, with a median age of 45 years old [60] and the extent of rehabilitation services provided, if any, was not reported.

The long-term sequelae of COVID-19 are well documented [61–63] however we found that none of the included studies followed up participants after the point of discharge and none of the studies reported on residual COVID-19 symptoms at the point of discharge or follow up. The long-term effect of multidisciplinary rehabilitation is unclear and remains to be investigated rigorously. Existing research in the older adult population indicates decline in function, increases in frailty and a reduction in quality of life over time following COVID-19 [64, 65]. It would be valuable to determine through robust experimental research if MDT rehabilitation can impact functional deterioration and worsening frailty over time in older adults with COVID-19 as it has been shown to benefit these outcomes with other older adult populations [66, 67].

This review included no studies reporting healthcare utilisation following MDT rehabilitation at the point of discharge or at follow up. It is important that intervention studies assess older adults’ healthcare use on discharge from acute or post-acute hospital settings for COVID-19 as people discharged from hospital following treatment for COVID-19 are at significantly higher risk for readmission to hospital when compared to demographically matched controls and people discharged from hospital following treatment for influenza, suggesting a significant burden to healthcare services for the cohort [68].

The 12 included studies in this review consisted of seven descriptive cohort studies, one pre-post intervention cohort, one quasi experimental study and three case series highlighting a dearth of robust experimental studies or analytical cohort studies describing the effect of multidisciplinary rehabilitation on the outcomes of older adults in the acute or post-acute setting following COVID-19 to facilitate systematic review and meta-analysis. A quasi-experimental study by Rodriguez and colleagues aimed to describe the effects of a multimodal rehabilitation programme in patients with COVID-19 admitted to the ICU [69] however this study was ineligible for inclusion in our review as the average age of the intervention cohort was 56.5 years and it was unclear if the intervention was multidisciplinary in nature. A large number of descriptive cohort studies and case series were not included in this review reporting outcomes following MDT rehabilitation following COVID-19 as their focus was on a younger population [30, 52–59, 70, 71]. GRADE analysis of included studies showed very low certainty of evidence which limits the applicability of results and highlights the importance of future trial studies to determine the effect of rehabilitation for the cohort.

Three studies included in this review excluded patients with a diagnosis of delirium or dementia [43, 45, 46]. Older adults with COVID-19 commonly present with delirium on admission or during the course of their acute illness in hospital [72–74]. Additionally, older adults with an underlying cognitive impairment or dementia pathology are at higher risk of delirium incidence [75]. Existing evidence from studies with older adults not specific to COVID-19 supports the assertion that older adults with cognitive impairment can benefit from rehabilitation [76, 77]. Exclusion of those with cognitive impairment in rehabilitation research, limits the applicability of outcomes to a significant cohort of older adults seeking acute medical care for COVID-19.

The results of this review must be considered in the context of the global progress with the roll out of COVID-19 vaccination programmes. The European Centre for Disease Prevention and Control (ECDC) reports a total of 966,099,169 vaccination doses administered as of the 14^th^ of December 2022 [78]. The total number of people who have been vaccinated with at least one dose in the European Union is reported as 342,182,404 in the total population, representing 75.5% of the population [78]. It is established that mRNA COVID-19 vaccination greatly reduces the risk of mortality, disease progression, death and mechanical ventilation [79]. Our review included studies in which patients were recruited between March 2020 and December 2021 and therefore not all patients could have been vaccinated. Three studies were carried out during a time when vaccinations were available to older adults [46, 48, 49]. It is possible to deduce that as more people are vaccinated worldwide that fewer adults and older adults will require hospitalisation and rehabilitation. However, there are cases of unvaccinated cohorts internationally due to inequity in vaccine roll out with the WHO reporting only 25% of older adults have had a complete series of vaccines in lower income countries [80]. It has also been reported that COVID-19 patients infected with the Omicron variant have a lower risk of hospitalisation compared with patients infected with the Delta variant [81, 82]. It is possible that new variants will emerge with unknown associated admission rates to hospital.

Geriatric rehabilitation programmes for patients with COVID-19 require additional consideration for the physical environment, equipment, resources and staffing in order to minimise the impact of infection control measures on patient experience and outcomes [28]. The multi-organ involvement of COVID-19 requires an interdisciplinary approach to address the numerous complications associated with COVID-19 infection [83] provided by an interdisciplinary team including, Physicians, Nurses, Physiotherapists, Occupational Therapists, Dietitians, Speech and Language Therapists, Psychologists and Social Workers [28]. In this systematic review, each study met the criteria for MDT rehabilitation however team composition varied. PT, OT, SLT and Psychology were the most prevalent disciplines. Few studies reported Dietitians as part of the MDT despite the high prevalence of malnutrition in COVID-19 hospitalised patients [84, 85]. Heterogeneity of rehabilitation programmes and limited reporting of rehabilitation programmes were evident in this systematic review however seven papers described their rehabilitation programme in sufficient detail [41, 43–46, 49, 50]. It is recommended that geriatric rehabilitation for COVID-19 should address frailty, malnutrition, cognition, activities of daily living and participation, mood, pain and symptom management, retraining of mobility, strengthening exercises, psychological disturbances, and speech and swallow impairments with discharge planning to facilitate follow up to the appropriate primary care or specialist outpatient care setting [28]. None of the studies included in this review described a rehabilitation programme that addressed all of these domains.

### Strengths and limitations

The conduct and reporting of this systematic review was in accordance with the MOOSE guidelines [31]. The identification of suitable papers was completed with a standardised and reproducible search strategy and with clear inclusion and exclusion criteria. A PRISMA flow diagram was used to map the flow of information through the different phases of the review. Critical appraisal of included papers was completed using the CASP checklist for cohort studies and the JBI Critical Appraisal Tool to assess bias. GRADE analysis also assessed the quality of evidence.

A limitation of this review is the heterogeneity of rehabilitation programmes with limited reporting of the frequency, intensity, time and type of interventions. No trial studies were included in this review and critical appraisal of the studies included highlight quality deficits which limits the internal and external validity of the findings.

### Clinical and research Implications

This review highlights the need for experimental studies exploring the effect of multidisciplinary rehabilitation on older adults with COVID-19. The ethical challenge this poses to the research community must be considered however as experimental studies would place patients into control and experimental groups.

This review highlights the need for greater attention to long term follow up in studies with older adults post COVID-19 to assess function, ongoing symptoms, and healthcare utilisation to determine the long-term effect of multidisciplinary rehabilitation. Long term outcomes and ongoing symptoms should be explored objectively by measures designed for the population and health states in question such as the COVID-19 Yorkshire Rehabilitation Scale (C-19 YRS) [86] which is recommended by the United Kingdom’s National Health Service [87] and the National Institute for Health and Care Excellence [22].

Given the heterogeneity of rehabilitation programmes in this review, future experimental research should describe a defined and reproducible rehabilitation programme using the TIDieR checklist [88]. An economic evaluation of multidisciplinary rehabilitation in this population could explore the financial implications to our health care systems. It is estimated that COVID-19 rehabilitation costs twice that of other rehabilitation units due to the complexity of its presentation, the heterogenous complications and the infection control measures required [89] however exact figures do not exist.

## Conclusion

This review demonstrates that multidisciplinary rehabilitation may result in improved functional outcomes on discharge from acute or post-acute hospital settings for older adults with COVID-19. There is a need for robust and experimental research into the long-term effect of rehabilitation for older adults following COVID-19. Future research should comprehensively describe MDT rehabilitation in terms of disciplines involved and the intervention provided using a standardised method of reporting.

## Supplementary Information

**Additional file 1. **Meta-analyses Of Observational Studies in Epidemiology (MOOSE) checklist. checklist of items detailing how the research was performed and reported.

**Additional file 2.** Search terms. Description of search concepts, synonyms and Boolean logic used.

## Data Availability

The authors declare that the data supporting the findings of this study are available within the article and its supplementary information files.

## Abbreviations

6MWT: Six Minute Walk Test
ADL: Activities of daily living
BI: Barthel Index
C-19 YRS: COVID-19 Yorkshire Rehabilitation Scale
CASP: Critical appraisal skills programme
CI: Confidence interval
ECDC: European Centre for Disease Prevention and Control
EuGMS: European Geriatric Medicine Society
FAC: Functional Ambulation Category
FEM: Fixed effects model
FIM: Functional independence measure
HSCP: Health and social care practitioner
ICU: Intensive Care Unit
IQR: Interquartile range
IRF PAI: Inpatient Rehabilitation Facility Patient Assessment Instrument
LOS: Length of stay
mBI: Modified Barthel Index
MDT: Multidisciplinary team
NICE: National Institute for Health and Care Excellence
NR: Not reported
OT: Occupational Therapy
PT: Physiotherapy
QOL: Quality of life
REM: Random effects model
SE: Standard error
SLT: Speech and language therapy
SMD: Standardised mean difference
WHO: World Health Organisation
