# Supplementary material for: Multidisciplinary inpatient rehabilitation for older adults with COVID-19: a systematic review and meta-analysis of clinical and process outcomes
Source: BMC Geriatr. 2023 Jun 27;23:391. doi: 10.1186/s12877-023-04098-4 (PMC10294371; doi:10.1186/s12877-023-04098-4)
Supplement: Supplementary file 2 — Additional file 2. Search terms. Description of search concepts, synonyms and Boolean logic used. [file 12877_2023_4098_MOESM2_ESM.docx]

**Additional file 2: Search terms**

|  | **AND** | **AND** | **NOT** |
| --- | --- | --- | --- |
| **Covid 19** | **rehabilitation** | **Hospital** | **children** |
| COVID  OR  COVID19  OR  "SARS‐CoV‐2"  OR  "SARS‐CoV2"  OR  SARSCoV2  OR  "SARSCoV‐2"  OR  "SARS coronavirus 2" OR  "2019 nCoV"  OR  "2019nCoV"  OR  "2019‐novel CoV"  OR  "nCov 2019" OR  "nCov 19" | rehabilitation  OR  “interprofessional rehabilitation”  OR  “multidisciplinary team”  OR  MDT  OR  physiotherap*  OR  “physical therap*”  OR  PT  OR  “speech and Language therap*”  OR  “speech and language patholog*”  OR  SLT  OR  SLP  OR  “occupational therap*”  OR  OT | hospital  OR  hospitalization  OR  hospitalisation  OR  inpatient  OR  “acute care”  OR  acute  OR  ward  OR  ICU  OR  “intensive care” OR  “critical care” | child*  OR  adolescent*  OR  adolescence  OR  youth  OR  teenage* |
